# Is reusing text from a protocol in the completed systematic review acceptable?

**DOI:** 10.1186/s13643-021-01675-9

**Published:** 2021-05-03

**Authors:** Dawid Pieper, Long Ge, Ahmed Abou-Setta

**Affiliations:** 1 Institute for Research in Operative Medicine, Faculty of Health, School of Medicine, Witten/Herdecke University, Ostmerheimer Str. 200, 51109 Cologne, Germany; 2 Evidence Based Social Science Research Center, School of Public Health, Lanzhou University, GRADE Chinese Center, Lanzhou, China; 3 George & Fay Yee Center for Healthcare Innovation, University of Manitoba, 753 McDermot Avenue, Winnipeg, MB R3E 0 T6 Canada; 4 Department of Community Health Sciences, University of Manitoba, Winnipeg, Canada

**Keywords:** Systematic review, Meta-analysis, Registration, Protocol, Plagiarism, Publishing

## Abstract

Published protocols have the potential to reduce bias in the conduct and reporting of systematic reviews (SR). When reporting the results of a completed SR, the question might arise whether text used in the protocol can also be used in the completed SR? Does this constitute text recycling, plagiarism, or even copyright infringement? In theory, no major changes to the protocol will be expected for the introduction and methods sections if the SR is completed in time. The benefits of maintaining the introduction and methods section of a protocol in the published SR are straightforward. Authors will require less time for writing up the completed SR. Potential benefits can also be expected for peer reviewers and editors. However, reusing text can be described as self-plagiarism. The question to be answered is whether this type of self-plagiarism is acceptable when copying text used previously (as would be the case when copying text from the protocol and pasting it into the subsequent completed SR)? The “traditional answer” to this question is “yes” because authors should not get credit for one piece of work for more than one time unless the work is cited appropriately. In contrast, we propose that in this context, it seems to be fully acceptable from a scientific and ethical perspective. As such, authors should not be accused of plagiarism in this case, but rather be encouraged to be efficient. However, legal issues need to be taken into consideration (e.g., copyright). We hope to stimulate a discussion on this topic among authors, readers, editors, and publishers.

## Background

Broadly speaking, a protocol should reduce the potential for bias in the conduct and reporting of a systematic review (SR), minimizing post hoc decisions driven by results and “gut feelings” about data analysis. Currently used critical appraisal tools to assess SRs also ask for that. For example, the risk of bias in systematic reviews (ROBIS) tool asks whether the review adhered to pre-defined objectives and eligibility criteria, and whether all predefined analyses were followed, or any departures explained [1].

The idea of publishing protocols irrespective of the underlying study design was already mentioned more than 50 years ago [2]. There is now a guideline for reporting SR protocols—PRISMA-P [3] and many journals now publish protocols, including SR protocols, at an increasing rate [4]. Besides publishing a protocol, authors might wish to make it available in a preprint server, institutional repository, or other platforms (e.g., Open Science Framework).

Once the protocol is published or made available, the issue of reusing the text might arise. When reporting the results of a completed SR, there is the question of what to do with descriptions of the introduction and methods already reported in the protocol. Can this text be used for drafting the completed SR? Does this constitute text recycling, plagiarism, or even copyright infringement? Editors at BioMed Central have produced a first draft of a guideline for text recycling that is open for discussion [5]. However, they do not specifically mention protocols and subsequent SRs.

## Main text

In theory, no major changes to the protocol stage will be expected for SRs. Changes to the introduction and methods section are more likely the longer the SR takes. In one analysis of 80 SRs, the median time from final literature search to journal submission was approximately 0.9 years for non-Cochrane SRs with published protocols [6]. When following 576 published Cochrane review protocols, the median time to publication of the full review was 2.8 years [7]. Although both are different measures, this highlights that there is a certain period of time where things can change. As a general rule, it can be assumed that the shorter the time period between completing the protocol and the full SR, the smaller is the likelihood that changes to the introduction will be needed to be made, as the evidence base, rationale and objectives should remain the same. However, there might be examples where amendments or changes will be necessary. An up-to-date example is the current COVID-19 pandemic when new study results are published daily. Thus, it will be very likely that changes to the introduction will be needed. Authors might also consider updating epidemiological data such as prevalence or incidence. Even if changes or amendments to the introduction are necessary, it is very unlikely that the whole introduction needs to be re-written but only parts of it.

Given that the methods are established a priori, the methods section of the protocol is typically written in the future tense. Assuming that all planned methods for the SR were followed as established a priori the main difference between the methods section of the protocol and the SR should be the tense. However, in practice, it might be rather rare for no changes to occur. Minor changes as well as specifications should be clearly reported as protocol amendments. Major differences can also occur due to advances in research methodology (e.g., new risk of bias tool and GRADE approach); however, this would be a very rare event. Any differences occurring between methods stated in the protocol and those used to produce the SR findings need to be made explicit [8]. In addition, any change to the protocol should also be explained. Otherwise, the SR will remain to be at risk of bias. Important to note that the update of the PRISMA guideline will use the term protocol amendments instead of deviations [9].

In summary, we argue that all elements of the introduction and methods section might be duplicated if no changes to the introduction and the methods section are necessary except changes to the tense. In particular, changes to the methods section can even be potentially harmful. If no changes in the methods between the protocol and review stage occurred, rewriting the methods section in the review might lead readers to believe (non-declared) post protocol changes were made because of not using the same wording.

### Benefits

The benefits of maintaining the introduction and methods section of a protocol in the published SR are straightforward. Authors will require less time for writing up the completed SR. This saving of time is not only about the time needed to draft each section, but also about omitting discussions and finding consensus with co-authors. If the protocol is published and has undergone peer review, one could argue that this content does not need to undergo full peer review again when submitting the full SR and thereby expediting the review process. It should be noted that this will only hold true for published protocols and for protocols without or only minor deviations from the protocol. Summing up the potential benefits for authors, peer reviewers, and editors, it is likely that they will result in faster publication times and less editorial burden.

### Challenges

Probably the biggest challenge around reusing the introduction and methods text is the perception and reality of plagiarism and self-plagiarism. There does not seem to be a clear-cut answer to this issue, and it might depend on the context. In Cochrane reviews, for example, this might not be an issue as the protocol and the completed SR are operated and handled within one organization (i.e., Cochrane). There is always a clear and obvious connection between the protocol and the completed SR. This could also be the case when submitting the completed SR to the same journal that already published the corresponding protocol (what in fact is the same as with the Cochrane Database of Systematic Reviews).

The idea of Registered Reports is quite different from traditional ways of publishing. The first part of a Registered Report is the protocol (stage 1) that will undergo peer review, while the second part is the subsequent completed study (stage 2) that will also undergo peer review. After having successfully passed stage 1, the authors are given the promise that their subsequent completed study will be accepted when following their protocol. In other words, the authors receive an in-principle acceptance for the completed study. Probably, the first journal that introduced this approach was *The Lancet* in 1997 [10]. In those days, they named the concept Protocol Reviews. Nowadays, the term Registered Reports describing the same concept has become more popular.

Needless to state, that other journals’ criteria for publication also need to be met after receiving an in-principle acceptance. Authors might also be asked to register their research in a recognized repository [11]. The Center for Open Science lists 275 journals (December 2020) using the Registered Reports publication format [12].

There is currently a paucity of general medical journals publishing protocols of SRs outside of organizations such as Cochrane or JBI, BMJ Open and BMC Systematic Reviews being among the most frequent [6]. To complicate matters, for the dissemination of the subsequent completed SR, authors might wish to disseminate their findings to a different journal audience. In this case, the link would be broken as protocols and subsequent SRs would be published in different journals, and maybe even with a different publisher. The idea of electronic threading or linking to overcome this is not new, but still needs to be implemented [13]. This might raise questions whether there might be legal issues about using former texts if they are not published via open access. whereby the authors maintain the copyright of the published material.

The question to be answered is whether this type of self-plagiarism is acceptable when copying text used previously (as would be the case when copying text from the protocol and pasting it into the subsequent completed SR)? The “traditional answer” to this question is yes because authors should not get credit for one piece of work for more than one time unless the work is cited appropriately. While we acknowledge that the general answer will be true in many instances, we believe that the answer to this question might depend on the context. In particular, in the context of protocols and subsequent SRs based on them using the former text from the protocol seems to be fully acceptable from a scientific and ethical perspective. Needless to state that SR authors should make this clear if they are using text from their protocol when reporting their completed review. However, one might argue that there is currently not a good way to do so. The authors should cite their protocol, of course. Linking protocols to reviews is also highly desirable. For example, for all open access papers indexed in PubMed Central, the protocols submitted to protocols.io (www.protocols.io) will automatically be linked to the full review report. However, there seems to be no accepted way of how to explicitly report which parts of the protocol have been copied, and which not. More work on these issues is highly appreciated.

A further challenge that can accompany this is that of authorship as authors can change between the protocol and the review. How to handle authorship changes from the protocol to the completed review? Are the authors allowed in this case to still use the text in the protocol verbatim? These questions occur and we do not have good answers to them yet.

A potential risk of copying the text from the protocol into the SR is that this will be done automatically. This could confound authors from thinking and reporting what was really done (or documenting protocol amendments). In this case, SRs would perfectly match to their protocols and lead one to believe that no deviations occur, while in fact, SRs would be reported inappropriately.

### Relevance to other study designs

Pros and cons are summarized in Table 1. In our commentary, we focused on SRs when discussing the benefits and challenges of maintaining the introduction and methods section of a protocol in a completed study. Nevertheless, our findings are not only applicable to SRs but to all study designs. It is only about the existence of a protocol. The idea of Registered Reports described above can be applied to all study designs, and even did not begin with SRs. Similarly, the issue of reusing text is also relevant for generic protocols, standard templates, updates of SRs, or living SRs. In all of these examples, the question to resolve is who owns the words.

**Table 1:** Pros and cons of reusing text from a protocol in the completed systematic review

Pros	Cons
Saves time	Perception and reality of plagiarism and self-plagiarism
Less burden for peer reviewers and editors	Clarification of authorships if authors are not the same at protocol and systematic review stage
Faster publication times	Risk of copying the text instead of reporting what was actually done

## Conclusion

With proper documentation, copying and pasting parts from the protocol into the subsequent SR seems to be acceptable to us as a time-saving exercise. When doing so, authors should not be accused of plagiarism. However, legal issues need to be taken into consideration. The regular use of Registered Reports as a new way of publishing can serve as a different way to sidestep this discussion. We hope to stimulate a discussion on this topic among authors, readers, editors, and publishers.

## Data Availability

Not applicable.

## Abbreviations

SR: Systematic review
JBI: Joanna Briggs Institute
